# Temporal patterns of radiocesium decline in current-year branches of coppiced *Quercus serrata* relative to stand age after the Fukushima nuclear accident

**DOI:** 10.1038/s41598-026-43819-8

**Published:** 2026-03-18

**Authors:** Wataru Sakashita, Satoru Miura, Eriko Ito, Junko Nagakura

**Affiliations:** 1 Center for Forest Restoration and Radioecology, Forestry and Forest Products Research Institute (FFPRI), 1 Matsunosato, Tsukuba, Ibaraki 305-8687 Japan; 2 Department of Forest Soils, FFPRI, 1 Matsunosato, Tsukuba, Ibaraki 305-8687 Japan; 3 Kansai Research Center, FFPRI, 68 Nagaikyutaroh, Momoyama, Fushimi, Kyoto, 612-0855 Japan

**Keywords:** Radiocesium, Current-year shoot, Coppiced konara oak, Stand age, Juvenile stage, Ecology, Ecology, Environmental sciences

## Abstract

Forest contamination by radiocesium (^137^Cs) has hindered the resumption of forestry activities after the Fukushima nuclear accident, particularly the production of coppiced konara oak (*Quercus serrata*) bed logs for shiitake mushroom (*Lentinula edodes*) cultivation. Methods are being developed to estimate stem ^137^Cs activity concentrations using current-year branches as an indirect indicator that can be used to identify available bed logs efficiently. However, the behavior of ^137^Cs in current-year branches—especially in the juvenile stage—is insufficiently understood, limiting efforts to improve the accuracy of stem concentration estimates. In this study, current-year branches were collected in the winters of 2020–2021 and 2025 from the same konara oak trees in 20 survey plots in Miyakoji, Tamura City, Fukushima Prefecture, where coppice regeneration occurred between 2011 and 2016. Temporal changes were examined using the abovementioned sampling data, along with the previous monitoring data from the winter of 2016–2017. Results showed that in konara oaks aged 1–3 years at the beginning of each 4-year interval, ^137^Cs activity concentrations in current-year branches decreased over four years by more than that expected from physical decay alone. By contrast, for trees aged 4–9 years at the start of the interval, the 4-year decline in concentrations was approximately consistent with physical decay. Thus, for konara oaks with a stand age of 4–9 years, the influence of stand age can be ignored when applying the developing relationship between current-year branch and stem ^137^Cs activity concentrations.

**Supplementary Information:**

The online version contains supplementary material available at 10.1038/s41598-026-43819-8.

## Introduction

In March 2026, fifteen years will have passed since the Fukushima Daiichi Nuclear Power Plant (FDNPP) accident, which contaminated forests—particularly those in Fukushima Prefecture—with radiocesium (^137^Cs). In the years immediately following the accident, the ^137^Cs distribution within forest ecosystems changed considerably^1–3^. Nonetheless, the current behavior of ^137^Cs in forests has generally stabilized^4–8^, with most of the ^137^Cs now being retained in the surface mineral soil horizon^7,9–12^.

Despite this stabilization, forest contamination by ^137^Cs continues to hinder the post-FDNPP resumption of forestry activities. Before the accident, coppiced konara oak trees (*Quercus serrata*) in Fukushima Prefecture were widely used as bed logs for shiitake mushroom (*Lentinula edodes*) cultivation^13^. However, bed-log production was suspended after deciduous broad-leaved forests became contaminated with ^137^Cs. To ensure that the ^137^Cs activity concentration in cultivated mushrooms does not exceed the food safety limit of 100 Bq/kg, the Forestry Agency^14^ established a threshold of 50 Bq/kg in bed logs. The ability to identify konara oak stems with ^137^Cs activity concentrations below this threshold may facilitate resumption of the use of contaminated deciduous broad-leaved forests.

Researchers have examined indirect indicators to enable efficient, nondestructive estimation of ^137^Cs activity concentrations in stems, which comprise bark, sapwood, and—in mature individuals—heartwood. Previous studies have found that ^137^Cs activity concentrations in the aboveground tissues of leaves or branches are strongly correlated with, and are thus reliable proxies of, ^137^Cs levels in stems^15–17^. Researchers have also evaluated seasonal fluctuations in ^137^Cs activity concentrations associated with the phenology of branches and leaves, recommending that tissue sampling for estimating stem concentrations should be conducted outside the shoot-flushing stage^18,19^. Furthermore, research suggests that obtaining a representative value of ^137^Cs activity concentrations in current-year branches within an area of several tens of meters on a side requires samples from five konara oak trees within that area^20^. However, as noted by Ohashi et al.^17^, changes in ^137^Cs activity concentrations in current-year branches from the juvenile stage—defined in the current study as the ages of 1–10 years—to the harvest stage (approximately 20 years old^11^) have not been evaluated despite the importance of understanding these changes for accurately estimating stem concentrations at harvest. Only a few studies have examined changes in ^137^Cs activity concentrations in coppiced konara oak shoots during the first few years after the juvenile stage^21^, and no studies have assessed these changes over longer-term periods. Even studies conducted after the Chernobyl accident lack data on young stands—particularly those aged 1–10 years—for deciduous broad-leaved trees, and most previous studies focus on conifers^22,23^. This issue should be addressed by continuously collecting current-year branches from the same konara oak trees of various young-stand ages over extended periods and evaluating the long-term changes in their ^137^Cs activity concentrations.

In this study, the monitoring of ^137^Cs activity concentrations began in the winter of 2016–2017^20,24^. Current-year branches were subsequently collected from the same konara oak trees in the winters of 2020–2021 and 2025, approximately four and eight years after the start of monitoring, respectively. By targeting coppiced konara oaks that were 1–6 years old in 2016, this study aimed to assess how stand age during the juvenile stage influences ^137^Cs activity concentrations in current-year branches. Building on this framework, this study further aimed to determine the stand age range during which current-year branches can be used to reliably estimate stem ^137^Cs activity concentrations.

## Materials and methods

### Study sites, sampling procedures, and ^137^Cs activity measurements

**Fig. 1:**
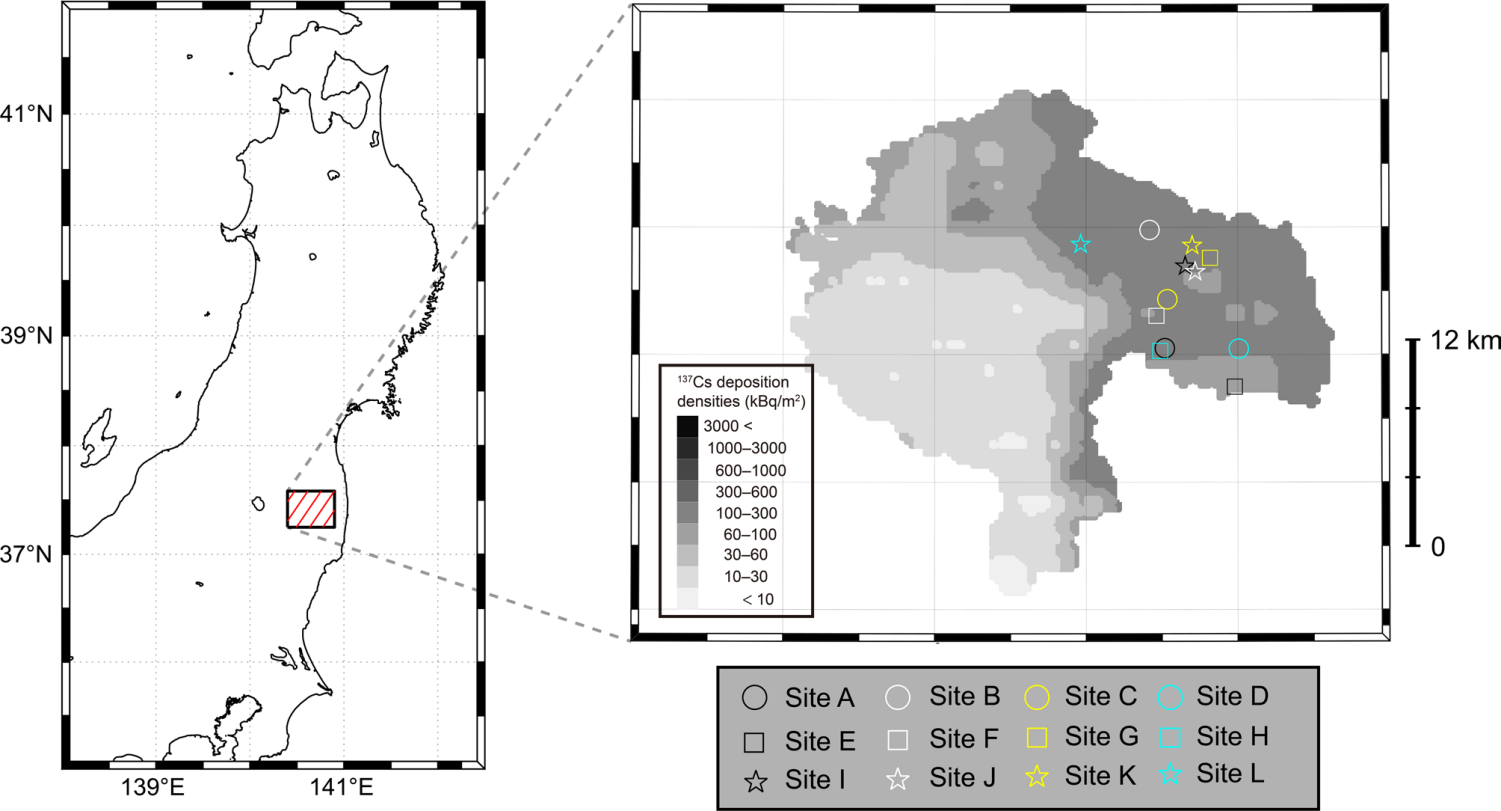
Studied sampling sites. The grayscale map in the zoomed-in image depicts the spatial distribution of ^137^Cs deposition—decay corrected to December 28, 2012—based on data from the sixth airborne monitoring survey^25^. The maps were generated using MATLAB with M_Map (version 1.4k)^26^.

This study was conducted in twelve deciduous broad-leaved forest stands in Miyakoji, Tamura City, Fukushima Prefecture (sites A–L in Fig. 1). One to three survey plots were established at each study site (Table 1), yielding 20 plots in total. These plots covered areas on the order of several tens of meters on a side and represented a subset of previously investigated plots^20,24^. Konara oak (*Q. serrata*) was the dominant tree species in all plots, and each plot underwent coppice regeneration between 2011 and 2016 (Table 1).

**Table 1:** Information about study sites. Latitude and longitude indicate the representative location of each study site.

Site name	Latitude	Longitude	Plot ID	Local place name	Year of coppicing	Stand age in 2016
A	N37°24′12″	E140°45′07″	A-1* A-2* A-3	Ohdaira-1-upper Ohdaira-1-lower Ohdaira-2	2016 2016 2013	1-year-old 1-year-old 4-year-old
B	N37°27′55″	E140°44′31″	B	Tokorokubo	2015	2-year-old
C	N37°25′44″	E140°45′13″	C	Yamaguchi	2015	2-year-old
D	N37°24′11″	E140°48′02″	D	Tochinokisawa	2014	3-year-old
E	N37°22′59″	E140°47′53″	E	Ohkubo	2014	3-year-old
F	N37°25′13″	E140°44′47″	F-1 F-2	Ishibashi-1 Ishibashi-2	2014 2013	3-year-old 4-year-old
G	N37°27′02″	E140°46′54″	G	Higashiyachi	2014	3-year-old
H	N37°24′07″	E140°44′56″	H-1 H-2	Kuroujika-upper Kuroujika-lower	2013 2013	4-year-old 4-year-old
I	N37°26′47″	E140°45′55″	I-1 I-2 I-3	Maeharazawa-1 Maeharazawa-2 Maeharazawa-3	2012 2012 2012	5-year-old 5-year-old 5-year-old
J	N37°26′36″	E140°46′19″	J-1 J-2	Yobiishi-1 Yobiishi-2	2012 2011	5-year-old 6-year-old
K	N37°27′25″	E140°46′12″	K	Dounouchi	2011	6-year-old
L	N37°27′28″	E140°41′47″	L-1 L-2	Kitasaku-upper Kitasaku-lower	2011 2011	6-year-old 6-year-old

*Plots A-1 and A-2 were coppiced in 2016 and were therefore defined as 1-year-old stands in this study.

Current-year branches were collected from December 2020 to January 2021 and in January 2025. This sampling period was selected to minimize the influence of seasonal variations in the ^137^Cs activity concentration caused by tree phenology, as a previous study has reported that concentrations are relatively stable during November–April^18^. The previous datasets ^20,24^ were likewise collected during the dormant season in 2016–2017, ensuring seasonal consistency among datasets. Current-year branch samples were obtained from the crowns of five konara oak trees, identified by Dr. Miura, in each survey plot using a pole pruner. However, several trees in some survey plots died within the observation period, so there were years in which samples could not be collected from all five trees (Table S1 in Supplementary Information). The collected samples were finely cut and homogenized, dried at 75 °C for at least 72 h, and packed into U8 containers. We collected and used the branch samples in accordance with all relevant guidelines, with the cooperation of the Fukushima Chuo Forestry Cooperative and with permission from the relevant forest owners.

We quantified ^137^Cs activity concentrations at the Forestry and Forest Products Research Institute (FFPRI) using high-purity germanium detectors (GEM20-70, GWL-120-15-LB-AWT, GEM40P4-76, GEM-FX7025P4-ST; ORTEC, Oak Ridge, USA). The peak efficiency of the U8 container was determined using standard sources (MX033U8PP; Japan Radioisotope Association, Tokyo, Japan). Measurement times ranged from 1,800 s (30 min) to 235,000 s. The current-year branches collected from trees 3–5 in plot H-1 (Table S1) in January 2025 had counting errors of 10–20%, whereas all other measurements had counting errors of below 10%. The ^137^Cs activity concentrations in samples collected from December 2020 to January 2021 were decay corrected to December 1, 2020, and those of samples collected in January 2025 were decay corrected to December 1, 2024. The samples are stored at FFPRI after the ^137^Cs activity measurements.

### Assessment of stand-age effect

We evaluated the eight-year temporal variation from 2016 to 2024 using the ^137^Cs activity concentrations of the current-year branches. The data from December 2016 to March 2017, obtained in previous studies^20,24^, were decay-corrected to December 1, 2016. The data for 2024 were newly collected in January 2025 and decay-corrected to December 2024. Data for tree 1 in plot H-1 were unavailable in 2016. For samples with ^137^Cs activity concentrations below the detection limit (not detected, ND; trees 3–4 in plot H-1 and tree 1 in plot I-1 in 2016), detection-limit values were used in the analysis (Table S1). This treatment allows temporal comparison because ND indicates concentrations below the detection limit rather than true zero, although it may provide conservative estimates when evaluating increases over time.

For each study plot, we used the ^137^Cs activity concentration in the current-year branches gathered in December 2016 as the baseline and determined whether the activity concentrations had declined after four and eight years to levels equivalent to or lower than those expected from physical decay. Assuming the ^137^Cs activity concentration on December 1, 2016, was 100%, physical decay—with a ^137^Cs half-life of 30.2 years—should have reduced it to 91.2% by December 1, 2020, and to 83.2% by December 1, 2024. We calculated these percentages for each monitored tree and then determined, for the median value of each plot, whether it differed significantly from the reduction expected from physical decay. These median values were calculated using log-transformed percentages, and statistical significance was assessed based on the 95% confidence interval (CI) of the median. These CIs were calculated using the boxchart function in MATLAB.

Furthermore, we grouped the study plots by stand age. We defined the konara oak trees in plots A-1 and A-2 as 1 year old in 2016, as this was their year of coppicing. For each stand age, we quantified the four-year change in ^137^Cs activity concentration as percentages for the two intervals (2016–2020 and 2020–2024). The changes observed during these two four-year intervals were then pooled and analyzed together to examine age-related patterns in temporal trends.

## Results

**Fig. 2:**
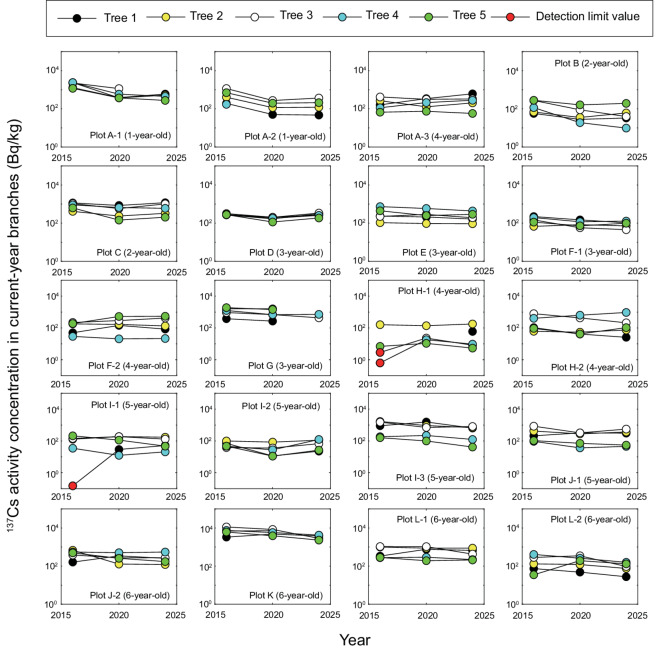
Eight-year temporal changes in ^137^Cs activity concentrations in current-year branches of *Q. serrata* (konara oak) in each study plot, measured at four-year intervals (December 2016^20,24^, December 2020, and December 2024). The ^137^Cs activity concentration data were decay corrected to December 1 of each year. The red circles indicate values at the detection limit; the ^137^Cs activity concentration in the current-year branches was below detectable levels. The numbers in parentheses indicate the stand age of each study plot in 2016.

**Fig. 3:**
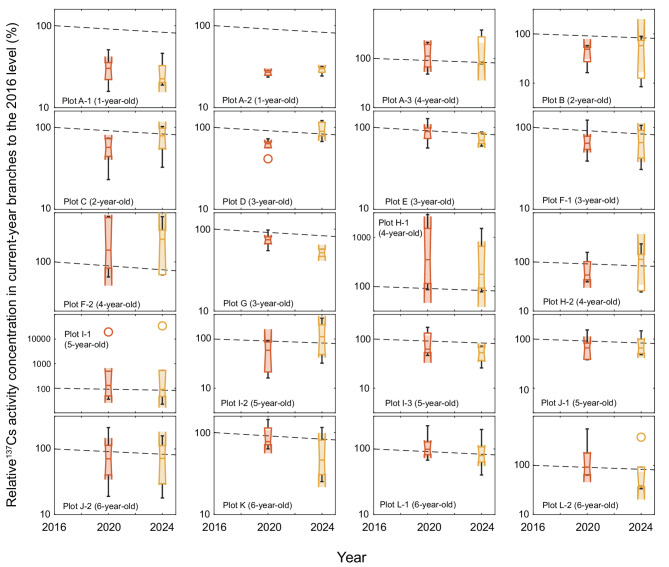
^137^Cs activity concentration in current-year branches relative to 2016 level (set as 100%) in each study plot. The dashed lines represent the reduction in ^137^Cs activity concentration caused by physical decay. The numbers in parentheses indicate the stand age of each study plot in 2016. The central line within each box represents the median, and the top and bottom edges indicate the 75th and 25th percentiles, respectively. The whiskers encompass values within 1.5 times the interquartile range, and the unfilled circles denote outliers. The shaded area illustrates the 95% CI around the median.

Figure 2 shows the changes in ^137^Cs activity concentrations in the current-year branches of coppiced konara oak from December 2016 to December 2024. It illustrates plot-specific temporal patterns and inter-tree variability; notably, in plots H-1 and I-1, some individuals showed concentrations below the detection limit in 2016 (two trees in H-1 and one tree in I-1), but these became measurable by 2020. Because absolute concentrations varied considerably among plots (Fig. 2), we further evaluated temporal changes using values normalized to 2016, as shown in Fig. 3.

Figure 3 presents the relative ^137^Cs activity concentrations in each study plot, normalized to 2016 levels. For the plots coppiced in 2016 (plots A-1 and A-2; 1 year old in 2016), the median relative concentrations in 2020 and 2024 were significantly lower than those expected from physical decay. In the plots coppiced in 2015 (plots B and C; 2 years old in 2016), the median decreased significantly in 2020 but not in 2024. For the plots coppiced in 2014 (3 years old in 2016), three patterns were observed: decreases in 2020 and 2024 consistent with physical decay (plot E), decreases exceeding those expected from physical decay in both years (plot G), and decreases of above those from physical decay only in 2020 (plots D and F-1). The plots coppiced in 2013 (plots A-3, F-2, H-1, and H-2; 4 years old in 2016) showed considerable individual variation with no significant differences, whereas those coppiced in 2011 (5 years old in 2016) and 2012 (6 years old in 2016) generally exhibited decreases according to physical decay, except for plots I-3 and L-2 in 2024. Overall, decreases exceeding those expected from physical decay were more frequently observed in plots that were younger in 2016, whereas older stands generally showed declines consistent with physical decay.

The ^137^Cs activity concentrations in current-year branches in several plots in 2020 and 2024 were substantially higher than those in 2016 (Fig. 3). For plot H-1, this was primarily because the ^137^Cs activity concentrations in two of the monitored konara oak trees in 2016 were below the detection limit (Fig. 2), with the small sample size (*n* = 4) further amplifying this effect. A similar tendency was also observed for the outlier in plot I-1.

**Fig. 4:**
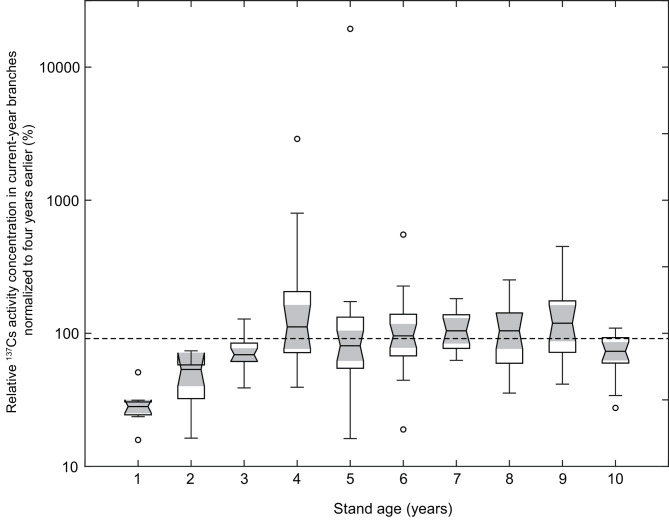
Relative ^137^Cs activity concentration in current-year branches, normalized to values of four years earlier, across stand ages. Because the two four-year intervals (2016–2020 and 2020–2024) were pooled, stand age is defined at the start of each interval; thus, trees that were 1–6 years old in 2016 correspond to 5–10 years old in 2020. The dashed line represents the expected value (91.2%) of the ^137^Cs activity concentration assuming physical decay over a four-year period. The central line within each box indicates the median, and the upper and lower edges represent the 75th and 25th percentiles, respectively. The whiskers extend to values within 1.5 times the interquartile range, and the unfilled circles denote outliers. The gray area illustrates the 95% CI around the median.

The relative ^137^Cs activity concentrations in the current-year branches, normalized to values from four years earlier, were evaluated by stand-age group (Fig. 4). Because the two four-year intervals were pooled, stand age was defined at the start of each interval; thus, trees that were 1–6 years old in 2016 correspond to 5–10 years old in 2020, which explains the presence of stand ages up to 10 years in Fig. 4. For trees that were 1 year old in 2016, the median relative ^137^Cs activity concentration in the current-year branches was 28.2% (95% CI: 25.1%–31.6%); for 2-year-old trees, 53.5% (95% CI: 40.1%–71.4%); and for 3-year-old trees, 69.0% (95% CI: 61.7%–77.1%). These values were significantly lower than the 91.2% expected from physical decay over four years. By contrast, among trees that were 4 years old or older, only the 10-year-old group showed an exceptionally low value (median: 75.0%, 95% CI: 63.1%–89.1%). The groups aged 4–9 years exhibited decreases consistent with those due to physical decay.

## Discussion

Our monitoring and analysis suggested that the ^137^Cs activity concentrations in the current-year branches of coppiced konara oaks aged 4 years or older decreased in accordance with physical decay. This finding may be explained by a reduced contribution of processes such as ^137^Cs translocation from the stump^21^ and growth-related dilution^27,28^, as discussed later, leading to concentrations that decline largely in accordance with physical decay in trees aged 4 years or older. Previous work^17^ established an empirical relationship between stem and current-year branch ^137^Cs activity concentrations and showed that this ratio can depend on stem diameter, while remaining relatively consistent for stems within a typical harvest-size range. Therefore, regarding previously proposed estimation methods for stem ^137^Cs activity concentrations^15,17^, sampling current-year branches from konara oaks more than four years after coppicing allows for accurate estimation of stem concentrations. However, since the 10-year-old konara oaks in this study showed greater declines in ^137^Cs activity concentrations than those expected from physical decay, further investigation is needed to determine whether this result also applies to trees aged 10 years or older.

For the konara oaks aged 1–3 years, the ^137^Cs activity concentrations in current-year branches decreased at rates exceeding those expected from physical decay. Thus, ^137^Cs activity concentrations in current-year branches tend to remain relatively high during the first few years after coppicing. Iwasawa^21^ reported a similar tendency—albeit not statistically significant—for ^137^Cs activity concentrations in the shoots and stems of coppiced konara oaks to decrease from approximately seven months after coppicing to one year later, which is consistent with the findings of the present study.

The reduction in ^137^Cs activity concentration in current-year branches during the juvenile stage to levels lower than those expected from physical decay may be driven by multiple factors. First, the higher ^137^Cs activity concentrations observed in the early juvenile stage (1–3 years old) could be attributed to the enhanced translocation of ^137^Cs initially stored in the stumps to the rapidly growing current-year branches^21^. Second, the rapid increase in biomass during the juvenile stage may have induced a dilution effect. Similar growth-driven dilutions have been reported to influence the seasonal variation of ^137^Cs activity concentrations in leaves and current-year branches^27,28^. In addition to these two factors, potential changes in root-mediated uptake during the juvenile stage cannot be excluded, although supporting evidence remains scarce and further investigation is required. The relative contributions of these factors can be assessed by monitoring changes in ^137^Cs distributions in the juvenile stage not only in shoots but also in stumps and roots.

Previous studies on the stand-age-related dynamics of ^137^Cs following the Chernobyl accident have largely focused on stands older than 10 years and mainly examined stems or foliage, whereas current-year shoots—often regarded as a minor pool of contamination—have been poorly addressed^22,23^. Post-Fukushima investigations have also mainly focused on observations conducted during the one to two years immediately following coppice regeneration^21^. Therefore, this study provides novel insights into the medium-term (1–10 years) dynamics of ^137^Cs in current-year branches of coppiced konara oaks and helps bridge this knowledge gap.

## Concluding remarks

This study is the first to document the dynamics of ^137^Cs activity concentrations in current-year branches of coppiced konara oaks across stand ages of 1–10 years in forests affected by either the Chernobyl or FDNPP accident. In konara oak stands aged 1–3 years in 2016, ^137^Cs activity concentrations in current-year branches tended to decline more rapidly than expected from physical decay alone relative to 2016. In stands aged 4–9 years at the beginning of each four-year interval (i.e., 2016 or 2020), the concentrations decreased in accordance with physical decay. To estimate stem ^137^Cs activity concentrations accurately using current-year branches, samples should be collected from coppiced konara oak stands that are at least 4 years old. These findings were effectively obtained using the study design, which involved the repeated monitoring of the same five konara oak trees in each survey plot and studying multiple plots for each stand age. This study design is essential for identifying the variation patterns of ^137^Cs activity concentrations within forest ecosystems, which exhibit inherently high spatial and temporal variabilities. The findings of this study contribute not only to improving estimates of stem ^137^Cs activity concentrations but also to validating models^29^ describing ^137^Cs dynamics during the juvenile stage of coppiced deciduous broad-leaved trees.

## Supplementary Information

Below is the link to the electronic supplementary material.

Supplementary Material 1

## Data Availability

All data used in this study are available in the Supplementary Information file (Table S1).
